# Food hypersensitivity: an examination of factors influencing symptoms and temporal changes in the prevalence of sensitization in an adult sample

**DOI:** 10.1038/s41430-023-01284-w

**Published:** 2023-03-24

**Authors:** Holly C. Y. Lam, Catherine Neukirch, Christer Janson, Judith Garcia-Aymerich, Michael Clausen, N. Sabrina Idrose, Pascal Demoly, Randi J. Bertelsen, Lidia C. Ruiz, Chantal Raherison, Deborah L. Jarvis

**Affiliations:** 1 National Heart and Lung Institute, Imperial College London, London, UK; 2 MRC Centre for Environment and Health, Imperial College London, London, UK; 3 Service de Pneumologie, AP-HP, Hôpital Bichat, Université de Paris, INSERM 1152, F-75018 Paris, France; 4 Department of Medical Sciences, Respiratory, Allergy and Sleep Research, Uppsala University, Uppsala, Sweden; 5 Barcelona Institute of Global Health (ISGlobal), Doctor Aiguader, 88, 08003 Barcelona, Spain; 6 Universitat Pompeu Fabra (UPF), Barcelona, Spain; 7 CIBER Epidemiología y Salud Pública (CIBERESP), Barcelona, Spain; 8 Children’s Hospital and Department of Allergy, Landspitali University Hospital, 101 Reykjavik, Iceland; 9 Allergy and Lung Health Unit, Melbourne School of Population and Global Health, The University of Melbourne, Carlton, VIC Australia; 10 Centre for Food and Allergy Research, Murdoch Children’s Research Institute, Parkville, VIC 3052 Australia; 11 Hôpital Arnaud de Villeneuve, University Hospital of Montpellier, Montpellier, France; 12 Department of Clinical Science, University of Bergen, Bergen, Norway; 13 Department of Occupational Medicine, Haukeland University Hospital, Bergen, Norway; 14 Social Epidemiology and Health Policy (SEHPO), Department of Family Medicine and Population Health (FAMPOP), University of Antwerp, Antwerp, Belgium; 15 University of Bordeaux, INSERM, BPH, U1219, F-33000 Bordeaux, France

**Keywords:** Risk factors, Biomarkers

## Abstract

**Background/Objectives:**

Food hypersensitivity (FHS) is common, but little is known about the factors associated with severe reactions, age of onset and whether sensitization persists. This study examines the factors associated with self-reported severe food reactions, onset age and the changes in prevalence of sensitization to foods over time in an adult sample.

**Subjects/Methods:**

We used data from adults taking part in the European Community Respiratory Health Survey (ECRHS) III (2010–2014) who provided information on food hypersensitivity, including symptoms, suspected culprit food and onset age (*n* = 4865). A subsample from six countries had serum food-specific IgE tested for 25 core foods and also in 10 years earlier (ECRHS II). We applied logistic regression and McNemar’s test for analyses.

**Results:**

The prevalence of self-reported FHS was 13.5% at ECRHS III. Of those providing information on symptoms (*n* = 611), 26.4% reported severe reactions. About 80% of 1033 reported food-specific reactions (reported by 596 participants) began after age 15. History of asthma (odds ratio OR 2.12 95% confidence interval CI 1.13–3.44) and a younger age of onset of FHS (OR 1.02, 95% CI 1.01–1.03, per year) were associated with higher risks of a lifetime experience of severe food reactions. In the subsample with IgE tested in both surveys (*n* = 1612), the overall prevalence of sensitization to foods did not change over 10 years.

**Conclusion:**

Our findings support previous observations of more severe food reactions in people with asthma and that most FHS reported by this sample started after age 15. We found no evidence of changes in the prevalence of sensitization to food in adults followed for 10 years.

## Introduction

Food hypersensitivity (FHS) refers to reproducible adverse reactions to food which clinically resemble allergy [1], covering food allergy and food intolerance. The prevalence of FHS has been estimated to be about 19.0% in the US [2] and 2–37% across Europe [3–5]. Although oral and skin symptoms were most commonly reported in Europe [4, 6–8], about one in five adults with self-reported FHS also reported that they had experienced respiratory or cardiovascular symptoms as part of their reaction to food in their lifetime [4]. One study in the US showed that 51.1% of adults with symptoms highly suggestive of food allergy had experienced at least one severe reaction during their lifetime [2].

Prediction of severe food reaction is difficult, as severity depends on a variety of food (e.g., allergens for food allergy) and host factors (such as sensitivity to allergens and severity of reactions to allergens) [9]. A prior clinical record of food reactions may predict severe reactions in the future, but a substantial proportion of people dying from food-related allergy had previously only presented with mild symptoms [9]. Better understanding of risk factors for severe food reactions may improve risk prediction and management. Previous studies have explored the risk factors of severe food allergic reactions, but some considered single outcome (e.g., only considered difficult breathing as outcome) [10], or severe cases only [11], and some reported contradicting results (some suggested specific-IgE level predicts severity of symptoms while some did not) [12, 13]. Current evidence suggests that pre-existing respiratory conditions such as wheezing and asthma were more common in those reporting breathlessness as a characteristic of their food reactions [10] and asthma was the main risk factor of food induced anaphylaxis and fatal food allergy [11].

Changes in sensitization to food with age [14] or over time in populations [15] have been reported in children, but little is known about new onset FHS or changes in sensitization to food in adults. A large population-based survey in the US showed that about half of adults with self-reported food reactions which highly suggested allergies had developed symptoms with ingestion to at least one food item after 17 years old [2]. A study using data from Sweden and Iceland (*n* = 807) reported the prevalence of sensitization to at least one food, and to peanut and soy in particular, decreased in adults over a 9-year period of follow-up even though the overall prevalence of reported FHS stayed the same [8]. However, this study only examined sensitization to six food items. There is little information on changes in the prevalence of sensitization to other food items in a wider adult population.

This study aims to improve the understanding of the characteristics of those with severe reactions following food ingestion by considering a wider range of health outcomes and population (i.e., allows comparisons between severe and less severe cases). This study also supplements the evidence on the changes in the prevalence of sensitization to the most common allergic food items among adults over time. Using information from a survey, conducted in a sample of the general adult population in Europe and Australia and collected as part of the European Community Respiratory Health Survey (ECRHS) III in 2010–2014, we identified the characteristics of participants who reported symptoms that were more likely to be associated with severe food reactions and examined the proportion of adult onset FHS (≥age 15). By linking information collected 10 earlier, we assessed the changes in prevalence of sensitization to food in this adult sample.

## Materials and methods

### Study participants

The ECRHS was established to examine the prevalence of asthma and identify its associated factors in European adult populations. The detailed study design was published elsewhere [16]. In brief, a community based random sample of 1500 men and 1500 women aged 20–44 years old were recruited from each participating center (55 centers from 19 countries) in 1991–1993 (ECRHS I). After the completion of a short postal questionnaire in the first stage, a “random“ sample of responders (300 men, 300 women randomly selected from the first stage sample) plus a sample about 150 of those reporting asthma symptoms in the postal questionnaire were invited for an interviewer-administered questionnaire and clinical assessment including venous sampling and skin prick test.

Two follow-ups were conducted for those who took part in the clinical assessment—ECRHS II (1998–2002) [17], and ECRHS III (2010–2014)—and these follow-ups included similar clinical assessments as ECRHS I. This analysis was restricted to the “random” sample of those who had took part in all follow-ups (ECRHS II to III, *n* = 5904, Supplementary Fig. 1) (see Appendix 1 in the Supplementary File for the number of participating centers and countries included for each analysis).

Informed consent was obtained from participants and ethics approval were granted from local ethics committees.

### Information collected

Questions on FHS collected in ECRHS III were used in this analysis. Participants were asked specifically about reaction to each of the 25 core foods (Supplementary Table 1) and were then asked to identify three foods (not necessarily from the core foods) that caused the “main problem” (ordered by the food giving the most severe reaction), the type of self-reported symptoms (from a pre-defined list as shown in Supplementary Table 2), the time from ingestion to reaction and the age of the first episode.

Serum specific IgE to food was tested in most participants of ECRHS II (related to availability of residual serum) and participants in six countries in ECRHS III (related to available funds, see Appendix 1 in Supplementary File for list of countries included). Venous blood samples were taken with serum stored at −20 °C before being tested in the laboratory (Kings College London in 2002 for ECRHS II, and AMC Amsterdam in 2013/2014 for ECRHS III) using the Pharmacia CAP System (Phadia, Uppsala, Sweden). Samples were screened against five food mix groups (excp1, expc2, excp3, fx5 and fx6) consisting of the 25 core food items (Supplementary Table 1) and if positive (sIgE level ≥0.35 ku/l), tested for positivity for individual foods within the food mix group.

Serum sIgE level to house dust mite, cat and Timothy grass pollen were assessed using the Pharmacia CAP system in ECRHS II and III. Sensitization to birch was examined using skin prick test in ECRHS III (reagents and standard lancets from ALK-ABELLO. Refer to Appendix 2 in Supplementary File for details). A summary of ECRHS data used in this analysis is summarized in Supplementary Table 3.

### Definitions used

FHS—a positive response to both questions “Have you ever had an illness or trouble caused by eating a particular food or foods?” AND “Have you nearly always had the same illness or trouble after eating this type of food?” (In Switzerland, the second question was omitted. FHS is defined as a positive answer to the first question AND the report of reaction to at least one individual food).

Severe food reaction—there is no single definition of severe food reaction. We defined severe reaction in this analysis with reference to the guidelines for diagnosing anaphylaxis. Symptoms that started within 4 h after ingestion of a particular food item and including at least one of the following:

1. The presence of skin-mucosal symptoms (rash or itchy skin OR itching, tingling or swelling in mouth OR difficulty in swallowing) PLUS evidence of EITHER respiratory compromise (breathlessness) AND/OR a drop in blood pressure (dizziness and fainting) AND/OR severe gastrointestinal symptoms (diarrhea or vomiting) [18].
2. In the absence of skin symptoms EITHER, a drop in blood pressure (dizziness and fainting), AND/OR respiratory compromise AND/OR laryngeal involvement (breathlessness) [18].
3. The need for an “emergency injection”.

Mild food reaction—any other symptoms reported after ingestion of food.

Sensitization to food and grass, cat, house dust mite—specific IgE greater than 0.35 kU/l to the allergens.

Positive skin prick test to birch—wheal size ≥3 mm for birch and positive control with no reaction to the negative control.

Asthma/nasal and skin allergies—a positive response to any of the questions “Have you ever had asthma?”, “Do you have any nasal allergies, including hay fever?” and “Have you ever had eczema or any kind of skin allergy?”.

### Analysis

#### Associated factors of severe reactions

Using information at ECRHS III, we applied univariate analysis (chi-square test, Wilcoxon rank sum test or *t*-test, where appropriate) and multiple logistic regression to identify associations of severe food reactions with sex, age, self-reported asthma/allergic (nasal and skin) history, total IgE level and sensitization to common inhalant allergens.

#### Age of onset of food reactions

Each participant reported details of up to three food-specific reactions. We firstly reported the proportion of participants with symptoms beginning (to any food) ≤15 years old, then described the median onset age of commonly reported foods.

#### Sensitization to food and severe symptoms

At ECRHS III, a subsample of 1673 participants had serum specific IgE measured for all 25 core foods. Among those reported FHS, we compared the proportion of positive IgE to the food between participants did and did not report severe symptoms using chi-square test.

#### Change of prevalence of sensitization to food

Sixteen hundred and twelve participants had food-specific serum IgE and specific IgE to inhalant allergens measured at both ECRHS II and ECRHS III. McNemar’s test was used to compare the changes of the overall prevalence of sensitization (then by year-of-birth cohort for food). Wilcoxon signed-rank test was applied to compare the median of IgE level to food mix groups between ECRHS III and ECRHS II.

All statistical analyses were conducted using R 3.6.3 [19]. A significance level of 0.05 was adopted in this analysis.

## Results

### Sample

After excluding centers that did not ask the FHS questions (*n* = 871) and those who did not respond to the FHS questions (*n* = 168) in ECRHS III, 4865 participants between 38–67 years old (52.3% female) were included (see Supplementary Table 4 for detailed characteristics).

### Prevalence of self-reported FHS and severe reactions

The prevalence of FHS (to any food) was 13.5% (655/4865) with 9.4% reporting FHS to at least one of the 25 core foods (Table 1). The prevalence of FHS was similar across age groups but was higher among females (16.9% vs. 9.7%, *p* < 0.0005). Among the 25 core foods, cow’s milk (2.6%), Hazelnut (2.1%), apple (2.1%), kiwi fruit (1.9%) and shrimp or lobster (1.7%) were the most commonly reported food related to FHS. Oyster (0.67%) and strawberry (0.51%) were most commonly named among non-core foods. More details of prevalence (number) of FHS to core (non-core) foods are shown in Table 2 (Supplementary Table 5).

**Table 1:** Prevalence of self-reported food hypersensitivity (FHS) and the associated symptoms at ECRHS III (*N* = 4865).

	Number	% in all participants	
All self-reported FHS	655	13.5%	
FHS by country:			
Australia	55	23.4%	
Belgium	62	17.8%	
Estonia	15	11.6%	
France	124	11.4%	
Iceland	64	17.7%	
Norway	76	21.7%	
Spain	40	4.8%	
Sweden	160	20.1%	
Switzerland	18	3.9%	
UK	41	15.1%	
FHS by age group:			
<50 years	227	14.2%	
50–59 years	268	13.5%	
≥60 years	160	12.5%	
FHS by sex:			
Female	430	16.9%	
Male	225	9.7%	
			% in 655
FHS by food type:			
1. At least one of the 25 core foods	457	9.4%	69.8%
2. Any other foods only	188	3.9%	28.7%
3. Information not provided	10	0.2%	1.5%
Number of foods reacted to:			
1. One food item only	297	6.1%	45.3%
2. More than one food items	348	7.2%	53.1%
3. Information not provided	10	0.2%	1.5%
Reported symptoms in relation to food that caused the main problems	*N* = 611^a^		% in 611
1. Mild symptoms only	450		73.6%
- Rash or itchy skin	99		16.2%
- Diarrhea or vomiting	162		26.5%
- Runny of stuffy nose	40		6.5%
- severe headache	42		6.9%
- Itching, tingling or swelling in mouth	130		21.3%
- Difficulty in swallowing	41		6.7%
2. Severe symptoms	161		26.4%
- Skin-mucosal + gastrointestinal symptoms	55		9.0%
- Faintness	45		7.4%
- Breathlessness	81		13.3%
- Emergency injection required	58		9.5%
3 Information not provided	26		4.3%

*FHS* food hypersensitivity.

^a^Among the 655 reported FHS, 611 had reported details of symptoms.

**Table 2:** Prevalence of self-reported food hypersensitivity (FHS) and the number of participants who experienced severe symptoms.

Food item	Self-reported FHS (*N* = 4865)			
	*n* (prevalence %)		Those reporting any symptoms	Those reporting severe symptoms^a^
			*n*	*n* (% in those reporting symptoms)
1	Cow’s milk	128 (2.6%)	86	10 (11.63%)
2	Hazelnut	102 (2.1%)	72	17 (23.61%)
3	Apple	98 (2.1%)	58	9 (15.52%)
4	Kiwi Fruit	92 (1.9%)	56	14 (25.00%)
5	Shrimp or Lobster	80 (1.7%)	54	12 (22.22%)
6	Walnut	66 (1.4%)	17	3 (17.65%)
7	Peach	56 (1.2%)	18	5 (27.78%)
8	Wheat	56 (1.2%)	26	5 (19.23%)
9	Peanut	53 (1.1%)	23	5 (21.74%)
10	Fish	49 (1.0%)	35	17 (48.57%)
11	Hen’s eggs	34 (0.7%)	23	3 (13.04%)
12	Carrot	33 (0.7%)	13	3 (23.07%)
13	Tomato	33 (0.7%)	18	1 (5.56%)
14	Bananas	29 (0.6%)	19	2 (10.53%)
15	Soybean	23 (0.5%)	8	2 (25%)
16	Melon	21 (0.4%)	6	0 (0)
17	Celery	16 (0.3%)	7	5 (71.43%)
18	Lentils	14 (0.3%)	3	0 (0)
19	Buckwheat	14 (0.3%)	4	1 (25%)
20	Rice	13 (0.3%)	7	0 (0)
21	Corn	12 (0.2%)	2	0 (0)
22	Mustard seed	9 (0.2%)	3	1 (33.33%)
23	Sesame seed	7 (0.1%)	5	1 (20.00%)
24	Sunflower seed	6 (0.1%)	3	1 (33.33%)
25	Poppy seed	5 (0.1%)	1	0 (0)

Table shows results of the 25 core foods and is ranked by prevalence of food-specific hypersensitivity.

*FHS* food hypersensitivity.

^a^Severe: breathlessness, fainting or dizziness, emergency injection required or having both skin-mucosal and gastrointestinal symptoms.

Of the 655 participants with self-reported FHS, 611 provided details of symptoms and were included in the symptom analysis. About one in four participants with self-reported FHS (26.4% of 611) reported symptoms suggestive of severe reactions. Breathlessness was the most commonly reported severe reaction (13.3%), followed by requiring emergency injection (9.5%) and skin-mucosal plus gastrointestinal symptoms (9.0%) (Table 1).

### Age of onset of FHS

**Fig. 1:**
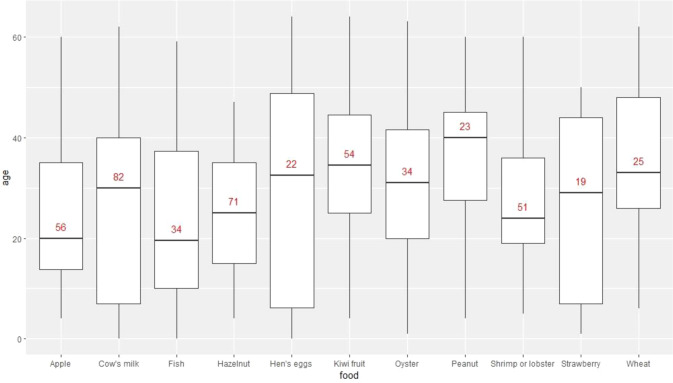
The age of first episode of the most commonly reported FHS cases by food. The number on the boxes indicates the number of participants included for each food.

Among the 596 participants reporting age of the first food reaction (any food), 76.5% had their first reaction at/after the age 15. These 596 participants reported a total of 1033 food-specific reactions (any food). About 81% of these 1033 were reported to have first occurred ≥15 years (78.0% for core food reactions). Among the most commonly reported foods, FHS triggered by fish had the lowest median age of first episode (20 years old, interquartile range IQR 10–37.2), while FHS related to peanut had the highest median age at 40 years old (IQR 27.5–45) (Fig. 1).

### Risk factors for severe reactions

In univariate analysis, self-reported history of asthma, nasal allergies and skin allergies, being sensitized to house dust mite (but not to cat, grass or birch) and having a younger age of first episode of FHS were associated with severe reactions (*p* ≤ 0.05) (Table 3).

**Table 3:** The associated factors of severity of symptoms (ECRHS III data).

	Self-report FHS	Symptoms (*n* = 611)		*p* value^b^ (Mild vs. Severe)	
		Mild^a^	Severe^a^		
				Univar^b^	Mult log^c^
Number of participants	655^d^	450	161		
Female, *n* (%)	430 (65.7%)	295 (65.56%)	101 (62.73%)	0.584	0.400
Mean age in year (SD)	53.4 (7.06)	53.74 (7.15)	53.12 (6.69)	0.571	
Country, *n* (%)				0.061	
Australia	55 (8.4%)	40 (8.89%)	12 (7.45%)		
Belgium	62 (9.5%)	43 (9.56%)	12 (7.45%)		0.967
Estonia	15 (2.3%)	12 (2.67%)	3 (1.86%)		0.604
France	124 (18.9%)	86 (19.11%)	33 (20.50%)		0.255
Iceland	64 (9.8%)	38 (8.44%)	23 (14.29%)		**0.025**
Norway	76 (11.6%)	57 (12.67%)	16 (9.94%)		0.494
Spain	40 (6.1%)	22 (4.89%)	18 (11.18%)		0.013
Sweden	160 (24.4%)	121 (26.89%)	34 (21.12%)		0.954
Switzerland	18 (2.8%)	–	–		–
UK	41 (6.3%)	31 (6.89%)	10 (6.21%)		0.761
Self-reported allergic history, *n* (%)					
Asthma	158 (24.1%)	87 (19.33%)	62 (38.51%)	<0.0005	**0.002**
Nasal allergies including hay fever	370 (56.5%)	251 (55.78%)	107 (66.46%)	0.012	0.163
Skin allergies, including eczema	379 (57.9%)	249 (55.33%)	109 (67.70%)	0.008	0.063
Total IgE level in kU/l, Median (IQR)	30.81 (11.28, 82.03)^e^	28.13 (10.93, 74.10)^f^	42.23 (13.57, 101.17)^g^	0.080	
Sensitization, *n* (%)					
House dust mite	100/541 (18.48%)	59/384 (15.36%)	36/134 (26.87%)	0.005	0.124
Cat	73/541 (13.49%)	48/384 (12.5%)	23/134 (17.16%)	0.228	
Grass	118/541 (21.81%)	83/384 (21.61%)	34/134 (25.37%)	0.478	
Birch	113/476 (23.74%)	83/337 (24.63%)	28/125 (22.40%)	0.707	
Age of first FHS to any food, Median (IQR)	25 (15, 40)^h^	26 (15, 40)	22 (10, 35)	0.001	**0.006**
Number of years since the first food reaction	26 (12, 38)	24 (11, 37)	30 (19, 41.5)	<0.005	

*FHS* food hypersensitivity, *IQR* interquartile range, *SD* standard deviation.

^a^Severe: breathlessness, fainting or dizziness, emergency injection required and have both skin-mucosal and gastrointestinal symptoms. Mild: rash or itchy skin, diarrhea or vomiting, runny or stuffy nose, severe headaches, itching, tingling or swelling in mouth, difficulty swallowing.

^b^Univariate tests: *t*-test for age; chi-square test for prevalence; Wilcoxon rank sum test for total IgE level and age of first FHS.

^c^Multiple logistic regression: factors with *p* < 0.05 in univariate tests were included. Sex and country were adjusted.

^d^Among the 655 had self-reported FHS, 44 did not provide details of symptoms.

^e^*n* = 541.

^f^*n* = 384.

^g^*n* = 134.

^h^*n* = 596.

Bold values identify statistical significance (*p* < 0.05).

After adjusting for sex, country and factors identified in univariate analysis, self-reported asthma (OR 2.12 95% confidence interval (CI) 1.13, 3.44) and younger age of first reaction to any food (OR 1.02, 95% CI 1.01, 1.03 per year) remained significantly associated with severe reactions (Table 3).

### Specific IgE

In ECRHS III, participants who had food-specific IgE measured (*n* = 1673) were slightly younger than those who did not have food-specific IgE measured (mean age 51.3 vs. 53.7 years old) but the prevalence of self-reported FHS was similar to that in the complete sample (14.4%).

The prevalence of sensitization to at least one of the five food mix groups was 14.8% (247/1673) (Supplementary Table 6). Among those sensitized and reported symptoms suggestive of FHS (*n* = 226), 69.5% (157/226) reporting mild reactions as defined (similar to that seen in the larger sample).

A total of 381 food-specific reactions were reported by these 1673 participants and 16.5% were sensitized to the relevant food (Supplementary Table 7). Among those with detailed symptom reported, the prevalence of sensitization to the relevant food was lower in those with severe reactions compared to those with milder reactions—but this difference, in this smaller sample, failed to reach conventional levels of statistical significance (6/54 vs. 39/183, *p* = 0.138) (Supplementary Table 7).

### Changes in prevalence of sensitization

The prevalence of sensitization to at least one food mix group was 14.5% at ECRHS II and 15.9% at ECRHS III (*n* = 1612). This change is not statistically significant, but subgroup analysis shows a significant increase, from 13.3 to 16.0% (*p* = 0.019), for the cohort 1954–1963 (Table 4).

**Table 4:** Prevalence of sensitization to a least one food at ECRHS II and III by birth cohort.

Birth cohort	*n*	Prevalence of sensitization to at least one food		*p* of McNemar’s test
		ECRHS II	ECRHS III	
All	1612	14.45%	15.88%	0.0849
1944–1953	163	18.40%	15.60%	0.4795
1954–1963	930	13.33%	16.02%	**0.019**
1964–1973	519	15.22%	15.60%	0.874

BOLD: *p* values < 0.05.

Prevalence of sensitization increased for food mix group epcx3 (banana, kiwi fruit, apple, peach, melon) from 9.3 to 12.0% (*p* = <0.0005) (Table 5). There were no significant changes in other food mix groups. The prevalence of sensitization to melon, a food item in epcx3, showed the largest increment among all core foods tested (0.7–2.6%), and although numbers were small, this was apparent in all six countries included (data not shown).

**Table 5:** Prevalence of sensitization to food and inhalant allergens at ECRHS II and ECRHS III.

	*n*	Prevalence of sensitization %		*p* values of McNemar’s test
		ECRHS II	ECRHS III	
Inhalant allergens	1612			
House dust mite		14.76	12.16	**<0.0005**
Timothy grass		15.51	14.27	**0.021**
Cat		9.18	7.57	**0.001**
Food mix groups	1612			
At least one group		14.45	15.88	0.085
Expc1: Hazelnut, walnut, celery, tomato, carrot		8.31	8.13	0.807
Expc2: Mustard, shrimp, sunflower seed, poppy seed, lentil		7.07	6.27	0.177
Expc3: Banana, kiwi, apple, peach, melon		9.31	11.97	<**0.0005**
Fx5: Cow’s milk, egg white, fish, soya bean, peanut, wheat		3.16	3.91	0.162
Fx6: Sesame, buckwheat, corn, rice		3.72	3.35	0.417
Food items				
Melon	1581	0.70	2.59	**<0.0005**
Lentils	1542	1.17	0.32	**0.006**
Hen’s egg	1606	0.50	1.31	**0.009**
Banana	1585	1.51	0.63	**0.011**
Peach	1556	3.15	1.80	**0.012**
Sunflower seed	1585	1.26	0.57	**0.022**
Wheat	1591	1.70	2.20	0.186
Cow’s milk	1606	0.68	1.12	0.211
Walnut	1593	1.44	1.04	0.211
Poppy seed	1586	0.88	0.57	0.228
Hazelnut	1587	5.48	4.91	0.233
Corn	1601	1.75	1.44	0.332
Shrimp or lobster	1583	3.72	3.22	0.332
Rice	1600	1.44	1.13	0.359
Soya	1606	1.25	0.93	0.359
Carrot	1589	2.14	1.95	0.710
Apple	1586	3.09	2.90	0.760
Buckwheat	1596	1.44	1.38	1
Celery	1587	2.33	2.33	1
Fish	1606	0.19	0.19	1
Kiwi fruit	1586	2.08	2.14	1
Mustard	1589	0.69	0.76	1
Peanut	1606	1.68	1.62	1
Sesame	1597	1.82	1.82	1
Tomato	1587	1.64	1.58	1

Food items are ranked by statistical significance of changes between the surveys (*p* value). BOLD: statistically significantly change in prevalence of sensitization between the two surveys (*p* < 0.05).

Median of IgE level to food mix group epcx1, epcx 2, epcx 3 and fx5 increased slightly from ECRHS II to III while IgE level to fx6 remained unchanged (Supplementary Table 8).

As has been previously reported in the entire ECRHS sample [20], we observed that the prevalence of IgE sensitization to house dust mite, grass and cat fell slightly between ECRHS II and ECRHS III (Table 5).

## Discussion

In this large multicentre epidemiological study based on self-reported symptoms and objective markers of IgE sensitization to some foods, we show that 13.5% of adults reported having FHS. Most of these reactions were triggered by one of 25 core foods previously identified and began at/after the age of 15 years. On the basis of self-reported symptoms and/or use of an injection occurring within 4 h of ingestion, we estimated that about one in four of this group had ever experienced a severe reaction, with severe reactions being more common in those with a younger age of onset and those with asthma. In this sample, there is little evidence that the overall prevalence of sensitization increased in 10 years.

Our analysis showed that self-reported history of asthma is associated with reporting severe reactions. This agrees with findings in previous studies that wheezing and asthma were associated with respiratory symptoms in food reactions [10] and that life-threatening manifestations of food anaphylaxis were usually caused by respiratory compromise [9].

We also found participants reporting an earlier age of onset of FHS were more likely to report symptoms that are suggestive of severe reactions. This may occur because our definition of FHS include food intolerance (rather than allergy) which is unlikely to be severe and is more common in adults [21]. The late onset of reaction to milk in Fig. 1 may reflect the development of intolerance in later life. The result may also have occurred because those with earlier onset have lived longer with their condition and therefore had more time to experience a severe reaction. However, when we looked at the association of age of onset with the presence of severe reactions within more narrow age groups (<50, 50–59, ≥60 years), the associations remained in each group with similar magnitude.

It is interesting to observe that FHS triggered by peanut had a median age of onset at 40 years, and that kiwi-related FHS also had a high median age of onset, in Fig. 1. These FHS cases may be linked to cross reactivity with pollen (e.g., sensitization to Bet-v1 like homologs, class 2 allergens) which may explain the less severe symptoms compared to those caused by other allergens of peanut (like stable storage proteins) that are more severe and usually begin in childhood [22].

Despite the minor increases in the median of IgE level of 4 out of 5 food mix groups, the overall prevalence of sensitization to food remained unchanged in the 10-year period across the 6 centers with available data. We are aware that one earlier report from the ECRHS looking at participants (*n* = 807) from only Iceland and Sweden suggested a 56% decrease in sensitization to food mix group fx5 between the first and second surveys [8]. We did not see a change in sensitization to fx5 when limiting our analysis to the Swedish and Icelandic samples over the study period (*n* = 550, result not shown). This is likely related to the different surveys considered (we compared the prevalence between ECRHS III and II while they compared ECRHS II and I) and that there is some between country variation in baseline prevalence [23].

To date, there are not many large studies reporting the proportion of severe food reactions, probably because of the discrepancies in defining symptoms and severity in different study centers, the difficulties in recruiting a representative sample and the issues in long-term follow-up. Our study included a large representative adult sample across Europe which could capture potential severe cases that were not presented in hospital or mortality data. The use of the same protocol across study sites reassures the consistency in measurements. The adoption of objective biomarkers and the diagnostic guideline of anaphylaxis for severe reactions will allow easy comparison with other studies. Our analysis also benefits from the 10-year dataset which allows the assessment of prevalence over time.

However, we acknowledged the use of self-reported symptoms and proxies for respiratory and cardiovascular symptoms does compromise the accuracy of outcome measurement. Recall bias and cohort effect may have reduced estimation accuracy. Biomarkers measured at survey times may not reflect the biological conditions at the time of food attacks. This, and the fact that self-reported FHS largely over-diagnosed proven IgE-dependent food allergy, may partly explain the low prevalence of sensitization to the food among those reported severe symptoms. Cohort effect detected should be considered with caution due to multiple testing. Finally, the power of food-specific analysis was also limited by sample size.

In conclusion, our study showed that one-quarter of the surveyed adults reporting FHS had reactions that can be linked to severe outcomes, with the history of asthma and a younger onset age of FHS as potential risk factors. Nearly 80% of the self-reported FHS in this adult sample developed at/after the age of 15 years old. There was no evidence of an increase in overall prevalence of sensitization to foods in this sample in the 10-year period.

## Supplementary information

Supplemental material

## Data Availability

The datasets generated during and/or analyzed during the current study are not publicly available due confidential agreement but are available from the corresponding author on reasonable request.
