# Supplementary material for: Food hypersensitivity: an examination of factors influencing symptoms and temporal changes in the prevalence of sensitization in an adult sample
Source: Eur J Clin Nutr. 2023 Mar 24;77(8):833–40. doi: 10.1038/s41430-023-01284-w (PMC10393775; doi:10.1038/s41430-023-01284-w)
Supplement: Supplementary file 1 — Supplemental material [file 41430_2023_1284_MOESM1_ESM.docx]

**Supplementary file**

**Title**

Food hypersensitivity: an examination of factors influencing symptoms and temporal changes in the prevalence of sensitization in an adult sample

**Authors**

Holly C. Y. Lam, PhD ^1,2^*, Catherine Neukirch, MD ^3^, Christer Janson, MD ^4^, Judith Garcia-Aymerich, MD^5,6,7^, Michael Clausen, MD ^8^, N. Sabrina Idrose, MSc ^9,10^, Pascal Demoly, MD ^11^, Randi J. Bertelsen, PhD ^12, 13^, Lidia C Ruiz, PhD ^14^, Chantal Raherison, MD ^15^, Deborah L. Jarvis, MD^1,2^

**Affiliations**

1 National Heart and Lung Institute, Imperial College, London, United Kingdom. (ching.lam@imperial.ac.uk; d.jarvis@imperial.ac.uk)

2 MRC Centre for Environment and Health, Imperial College London, London, United Kingdom.

3 Service de Pneumologie, AP-HP, Hôpital Bichat, Université de Paris, INSERM 1152 F-75018 Paris France. ([catherine.neukirch@aphp.fr](mailto:catherine.neukirch@aphp.fr))

4 Department of Medical Sciences, Respiratory, Allergy and Sleep Research, Uppsala university, Sweden. (christer.janson@medsci.uu.se)

5 Barcelona Institute of Global Health (ISGlobal), Doctor Aiguader, 88, 08003 Barcelona. ([judith.garcia@isglobal.org](mailto:judith.garcia@isglobal.org))

6 Universitat Pompeu Fabra (UPF), Barcelona, Spain

7 CIBER Epidemiología y Salud Pública (CIBERESP)

8 Children´s Hospital and Dept. of Allergy, Landspitali University Hospital, 101 Reykjavik, Iceland. ([mc@landspitali.is](mailto:mc@landspitali.is))

9 Allergy and Lung Health Unit, Melbourne School of Population and Global Health, The University of Melbourne, Carlton, VIC, Australia. ([sabrina.idrose@unimelb.edu.au](mailto:sabrina.idrose@unimelb.edu.au))

10 Centre for Food and Allergy Research, Murdoch Children’s Research Institute, Parkville, VIC, 3052, Australia ([sabrina.idrose@unimelb.edu.au](mailto:sabrina.idrose@unimelb.edu.au))

11 Hôpital Arnaud de Villeneuve, University Hospital of Montpellier, Montpellier, France. ([pascal.demoly@inserm.fr](mailto:pascal.demoly@inserm.fr))

12 Department of Clinical Science, University of Bergen, Bergen, Norway (Randi.J.Bertelsen@uib.no)

13 Department of Occupational Medicine, Haukeland University Hospital, Bergen Norway

14 Social Epidemiology and Health Policy (SEHPO), Department of Family Medicine and Population Health (FAMPOP), University of Antwerp ([Lidia.casas@uantwerpen.be](mailto:Lidia.casas@uantwerpen.be))

15 University of Bordeaux, INSERM, BPH, U1219, F-33000 Bordeaux, France (chantal.raherison@chu-guadeloupe.fr)

**Summary**

This supplementary file contains 2 appendices, 1 figure and 8 tables. The appendices give extra details of the method, Supplementary Figure 1 shows the participants selection process and the supplementary tables provide supportive information for the Results section.

**Appendix 1**

Number of Centres and Countries included

Participants in ECRHS III were from 26 centres in 12 countries (Australia, Belgium, Estonia, France, Germany, Iceland, Italy, Norway, Spain, Sweden, Switzerland and UK) but only 4865 participants from 21 centres in 10 countries (2 centres in Germany and three centres in Italy excluded) completed the FHS questions at ECRHS III. Prevalence of FHS, associated factors of severe reactions and onset age of FHS were examined in this sample.

At ECRHS III, a subsample of 1673 participants from 16 centres in six countries (in France, Iceland, Norway, Spain, Sweden and the UK) also had serum specific IgE measured for all 25 core foods. We applied logistic regression to compare the characteristics of participants (sex, age and self-reported asthma/allergic history) between those took part in serum sIgE test to food and those did not. Prevalence of sensitization and the association with severe food reactions were examined in this subsample.

The 1612 participants from six countries (France, Iceland, Norway, Spain, Sweden and the UK) had food specific serum IgE measured at both ECRHS II and ECRHS III were included for the assessment of changes in prevalence of sensitization between ECRHS II and III.

**Appendix 2**

Methods for skin prick test

Sensitization to birch was examined using skin prick test in ECRHS III (reagents and standard lancets from ALK-ABELLO). Birch solution at 10 HEP, a positive control (10mg/mL histamine) and a negative control (0.9% saline) were applied on participants’ forearm. The widest diameter and its perpendicular diameter were recorded.

**Supplementary Figure 1** Flow chart showing the selection of participants in analyses.

ECRHS III n=5904

(3536 dropped out and 867 rejoint)

1720 (29.1%) Conducted serum IgE test to food allergens

1673 conducted serum IgE test to all 25 food and answered to FHS questions

Sensitization analysis

ECRHS I n=14196

ECRHS II n=8573

(5623 dropped out)

4865 (82.4%) answered to FHS questions

FHS analysis

4043 (47.2%) conducted serum IgE test to food allergens

1612 conducted serum IgE test to food allergens in ECRHS II & III

Assessment of changes in sensitization level in 10 years

**Supplementary Table 1** Food mix groups for serum IgE test

| Food mix group | Food items |
| --- | --- |
| epcx1 | Hazelnut, walnut, celery, tomato, carrot |
| epcx2 | Mustard, shrimp or lobster, sunflower seed, poppy seed, lentil |
| epcx3 | Banana, kiwi fruit, apple, peach, melon |
| fx5 | Cow’s milk, hen’s egg white, cod fish, soya bean, peanut, wheat |
| fx6 | Sesame, buckwheat, corn, rice |

**Supplementary Table 2** Symptoms of food reactions listed in questionnaire

| Symptom group | Symptoms of reactions after ingestion of food |
| --- | --- |
| Skin-mucosa | A rash or itchy skin |
|  | Runny or study nose |
|  | Itching, tingling or swelling in the mouth, lips or throat |
|  | Difficulty swallowing; |
| Gastrointestinal | Diarrhoea or vomiting |
| Proxy of respiratory compromise | Breathlessness |
| Proxy of drop of blood pressure | Fainting or dizziness |
| Emergency injection | Symptoms so severe you had an emergency injection from a doctor or had use an epipen |
| Other | Severe headaches |

**Supplementary Table** 3 ECRHS data used in this analysis

| Study tool | ECRHS II | ECRHS III |
| --- | --- | --- |
| Questionnaire |  |  |
| -Self-reported FHS (any food) | Not used | Yes |
| -Self-reported FHS (25 food items) | NA | Yes |
| -Detailed symptoms and age of first food reaction | NA | Yes |
|  |  |  |
| Serum IgE test |  |  |
| -25 core foods | Yes | Yes |
| -Dust mite | Yes | Yes |
| -Cat | Yes | Yes |
| -Timothy grass | Yes | Yes |
|  |  |  |
| Skin prick test- birch | NA | Yes |
|  |  |  |

**Supplementary Table 4** Characteristics of participants included in food hypersensitivity (FHS) prevalence analysis at ECRHS III.

| Characteristics | Number (%) |
| --- | --- |
| Total number of participants | 4865 |
|  |  |
| Sex – Female | 2543 (52.3%) |
| Age (year) | Mean= 53. 7 (SD=7.1), range 38-67 |
|  |  |
| Country |  |
| Australia | 235 (4.8%) |
| Belgium | 348 (7.2%) |
| Estonia | 129 (2.7%) |
| France | 1086 (22.3%) |
| Iceland | 362 (7.4%) |
| Norway | 350 (7.2%) |
| Spain | 827 (17.0%) |
| Sweden | 798 (16.4%) |
| Switzerland | 458 (9.4%) |
| UK | 272 (5.6%) |
|  |  |
| Self-reported history of |  |
| Asthma | 684 (14.1%) |
| Nasal allergies including hay fever | 1582 (32.5%) |
| Skin allergies or eczema | 1966 (40.4%) |
|  |  |
| Participants with blood samples for total and inhalant allergen specific IgE tests | 3745 |
| Total IgE level in kU/L  Median (interquartile range, IQR) | 25.4 (10.3, 65.1) |
|  |  |
| Prevalence of sensitization^1^ to common inhalant allergens: |  |
| House dust mite | 485 (13.0%) |
| Cat | 290 (7.7%) |
| Timothy grass pollen | 495 (13.2%) |
| Birch pollen^2^ | 296/3345 (8.9%) |

1 Specific IgE level ≥ 0.35 kU/L in blood sample

2 Sensitization to birch pollen was conducted using skin prick test. Sensitization is defined as with wheal diameter≥3mm (with positive result in control and no reaction in negative control)

**Supplementary Table 5** Other foods that were commonly reported (n>10) to be associated with food hypersensitivity.

| Food | n |
| --- | --- |
| Oyster | 33 |
| Strawberry | 25 |
| Garlic | 19 |
| Onion | 19 |
| Pineapple | 18 |
| Blue Mussel | 16 |
| Cherry | 16 |
| Chocolate | 16 |
| Pear | 14 |
| Orange | 13 |
| Wine (not specified) | 13 |

**Supplementary Table 6** Prevalence of sensitization to the 5 food mix groups and the 25 foods tested (Specific IgE level ≥ 0.35 kU/L in blood sample) in participants included in FHS analysis at ECRHS III (N=1673). Food items in the table are ranked by overall prevalence of sensitization.

|  | Number of sensitized | Prevalence of sensitization % | | | | | | |
| --- | --- | --- | --- | --- | --- | --- | --- | --- |
|  |  | All  N=1673 | France  N=303 | Iceland  N=225 | Norway  N=225 | Spain  N=400 | Sweden  N=303 | UK  N=217 |
| Food mix group | | | | | | | | |
| At least one group | 247 | 14.76% | 14.52% | 11.56% | 15.56% | 13.75% | 18.15% | 14.75% |
| Expc1:  Hazelnut, walnut, celery, tomato, carrot | 120 | 7.17% | 7.26% | 5.33% | 9.78% | 3.00% | 11.22% | 8.29% |
| Expc2:  Mustard, shrimp, sunflower seed, poppy seed, lentil | 91 | 5.44% | 6.60% | 6.22% | 3.11% | 6.00% | 5.94% | 3.69% |
| Expc3:  Banana, kiwi, apple, peach, melon | 187 | 11.18% | 9.57% | 8.00% | 13.78% | 10.00% | 13.86% | 12.44% |
| Fx5:  Cow’s milk, egg white, fish, soya bean, peanut, wheat | 65 | 3.89% | 4.29% | 3.56% | 3.56% | 4.00% | 3.63% | 4.15% |
| Fx6: Sesame, buckwheat, corn, rice | 41 | 2.45% | 3.96% | 2.22% | 2.67% | 2.50% | 0.99% | 2.30% |
| Food item | | |  |  |  |  |  |  |
| At least one food item | 187 | 11.18% | 13.20% | 8.89% | 9.33% | 8.75% | 15.84% | 10.60% |
| Hazelnut | 91 | 5.44% | 5.94% | 2.22% | 8.00% | 1.25% | 10.23% | 6.45% |
| Shrimp or lobster | 58 | 3.47% | 5.28% | 3.56% | 1.78% | 3.75% | 4.62% | 0.46% |
| Apple | 57 | 3.41% | 4.29% | 1.78% | 3.56% | 1.50% | 4.29% | 5.99% |
| Melon | 56 | 3.35% | 4.62% | 0.89% | 2.22% | 2.00% | 4.95% | 5.53% |
| Celery | 48 | 2.87% | 3.96% | 2.67% | 1.78% | 1.25% | 2.97% | 5.53% |
| Kiwi fruit | 43 | 2.57% | 5.28% | 1.78% | 0.89% | 2.00% | 2.97% | 1.84% |
| Carrot | 40 | 2.39% | 3.63% | 1.33% | 0.89% | 1.75% | 1.98% | 5.07% |
| Peach | 39 | 2.33% | 2.97% | 3.56% | 1.33% | 1.75% | 2.97% | 1.38% |
| Tomato | 35 | 2.09% | 3.30% | 2.22% | 0.89% | 1.25% | 1.65% | 3.69% |
| Sesame seed | 29 | 1.73% | 3.63% | 2.22% | 0.89% | 1.50% | 0.66% | 1.38% |
| Wheat | 29 | 1.73% | 2.97% | 2.22% | 0.44% | 1.50% | 1.32% | 1.84% |
| Peanut | 28 | 1.67% | 2.97% | 1.78% | 0.44% | 1.00% | 1.65% | 2.30% |
| Buckwheat | 22 | 1.32% | 2.64% | 1.33% | 0.89% | 1.25% | 0.66% | 0.92% |
| Egg white | 22 | 1.32% | 1.65% | 1.78% | 1.33% | 1.00% | 0.99% | 1.38% |
| Cow's milk | 21 | 1.26% | 1.32% | 0.89% | 0.89% | 1.25% | 1.32% | 1.84% |
| Corn | 20 | 1.20% | 2.31% | 0.89% | 0.44% | 1.25% | 0.66% | 1.38% |
| Walnut | 20 | 1.20% | 2.31% | 0.89% | 0.44% | 1.25% | 0.66% | 1.38% |
| Rice | 19 | 1.14% | 2.31% | 0.89% | 0.44% | 1.25% | 0.66% | 0.92% |
| Mustard | 17 | 1.02% | 1.98% | 0.89% | 0.00% | 1.25% | 0.66% | 0.92% |
| Banana | 16 | 0.96% | 1.65% | 1.78% | 0.00% | 1.00% | 0.33% | 0.92% |
| Soya bean | 16 | 0.96% | 1.65% | 0.44% | 0.44% | 1.25% | 0.66% | 0.92% |
| Sunflower seed | 15 | 0.90% | 1.65% | 0.44% | 0.00% | 1.00% | 0.99% | 0.92% |
| Poppy seed | 14 | 0.84% | 1.65% | 0.44% | 0.00% | 1.00% | 0.66% | 0.92% |
| Lentil | 11 | 0.66% | 0.66% | 0.89% | 0.00% | 1.00% | 0.66% | 0.46% |
| Fish (Cod) | 3 | 0.18% | 0.33% | 0.00% | 0.00% | 0.50% | 0.00% | 0.00% |

**Supplementary Table 7** Prevalence of sensitization by severity of symptoms among those with serum IgE test for food in ECRHS III (n=1673). Table is ranked by the number of self-reported FHS.

| Food | Self-reported FHS | Prevalence of sensitization | | | |
| --- | --- | --- | --- | --- | --- |
|  | n (% in 1673) | Self-reported FHS | Any symptoms | Severe symptoms | Mild symptoms |
| Hazelnut | 49 (2.93%) | 27/49 | 23/37 | 3/10 | 20/27 |
| Apple | 36 (2.15%) | 12/36 | 9/23 | 1/3 | 8/20 |
| Cow's milk | 35 (2.09%) | 0/35 | 0/27 | 0/3 | 0/24 |
| Kiwi fruit | 35 (2.09%) | 5/35 | 3/24 | 0/3 | 3/21 |
| Shrimp or lobster | 27 (1.61%) | 3/27 | 2/22 | 1/7 | 1/15 |
| Wheat | 25 (1.49%) | 2/25 | 1/14 | 0/4 | 1/10 |
| Peanut | 23 (1.37%) | 1/23 | 1/13 | 0/5 | 1/8 |
| Walnut | 21 (1.26%) | 2/21 | 1/6 | 1/1 | 0/5 |
| Fish* | 20 (1.20%) | 0/20 | 0/17 | 0/11 | 0/6 |
| Peach | 18 (1.08%) | 0/18 | 0/7 | 0/1 | 0/6 |
| Tomato | 14 (0.84%) | 1/14 | 0/9 | 0/1 | 0/8 |
| Carrot | 10 (0.60%) | 3/10 | 2/4 | 0/1 | 2/3 |
| Bananas | 10 (0.60%) | 1/10 | 0/8 | -/0 | 0/8 |
| Soybean | 8 (0.48%) | 1/8 | 1/4 | -/0 | 1/4 |
| Hen’s eggs | 7 (0.42%) | 0/7 | 1/6 | -/0 | 1/6 |
| Melon | 7 (0.42%) | 1/7 | 0/1 | -/0 | 0/1 |
| Corn | 6 (0.36%) | 1/6 | 1/3 | -/0 | 1/3 |
| Buckwheat | 6 (0.36%) | 0/6 | 0/1 | -/0 | 0/1 |
| Rice | 5 (0.30%) | 1/5 | 0/3 | -/0 | 0/3 |
| Celery | 5 (0.30%) | 2/5 | 0/3 | 0/2 | 0/1 |
| Mustard | 4 (0.24%) | 0/4 | 0/2 | 0/1 | 0/1 |
| Lentils | 3 (0.18%) | 0/3 | 0/1 | -/0 | 0/1 |
| Poppy seed | 3 (0.18%) | 0/3 | -/0 | -/0 | -/0 |
| Sesame seed | 2 (0.12%) | 0/2 | 0/2 | 0/1 | 0/1 |
| Sunflower seed | 2 (0.12%) | 0/2 | -/0 | -/0 | -/0 |
| Total no. of FHS | 381 | 63/381(16.54%) | 45/237 (18.99%) | 6/54 (11.11%) | 39/183 (21.31%) |

*Serum IgE test was specific for cod fish.

FHS: food hypersensitivity

**Supplementary Table 8** Median and interquartile range (IQR) of specific IgE level to food mix groups at ECRHS II and III.

| Food mix group | Median (IQR) IgE level | | p-value of Wilcoxon signed rank test for  Median at ECRHS III vs. II |
| --- | --- | --- | --- |
| (N=1612) | ECRHS II | ECRHS III |  |
| Epcx1 | 0.03 (0.02, 0.05) | 0.05 (0.04, 0.07) | **<0.0005** |
| Epcx2 | 0.04 (0.02, 0.07) | 0.06 (0.04, 0.09) | **<0.0005** |
| Epcx3 | 0.06 (0.05, 0.11) | 0.14 (0.12, 0.20) | **<0.0005** |
| Fx5 | 0.04 (0.03, 0.07) | 0.05 (0.04, 0.09) | **<0.0005** |
| Fx6 | 0.11 (0.09, 0.14) | 0.11 (0.10, 0.14) | 0.691 |

**BOLD**: p-values < 0.05
